# Effects of Supplemental Oxygen on Cardiovascular and Respiratory Interactions by Extended Partial Directed Coherence in Idiopathic Pulmonary Fibrosis

**DOI:** 10.3389/fnetp.2022.834056

**Published:** 2022-03-15

**Authors:** Laura M. Santiago-Fuentes, Sonia Charleston-Villalobos, Ramón González-Camarena, Andreas Voss, Mayra E. Mejía-Avila, Ivette Buendía-Roldan, Sina Reulecke, Tomás Aljama-Corrales

**Affiliations:** ^1^ Electrical Engineering Department, Universidad Autónoma Metropolitana, Mexico City, Mexico; ^2^ Health Science Department, Universidad Autónoma Metropolitana, Mexico City, Mexico; ^3^ Institute of Biomedical Engineering and Informatics, University of Technology Ilmenau, Ilmenau, Germany; ^4^ National Institute of Respiratory Diseases, Mexico City, Mexico

**Keywords:** idiopathic pulmonary fibrosis, supplemental oxygen, systems interactions, time-frequency cardiorespiratory interactions, extended partial directed coherence

## Abstract

Idiopathic pulmonary fibrosis (IPF) is a chronic and restrictive disease characterized by fibrosis and inflammatory changes in lung tissue producing a reduction in diffusion capacity and leading to exertional chronic arterial hypoxemia and dyspnea. Furthermore, clinically, supplemental oxygen (SupplO_2_) has been prescribed to IPF patients to improve symptoms. However, the evidence about the benefits or disadvantages of oxygen supplementation is not conclusive. In addition, the impact of SupplO_2_ on the autonomic nervous system (ANS) regulation in respiratory diseases needs to be evaluated. In this study the interactions between cardiovascular and respiratory systems in IPF patients, during ambient air (AA) and SupplO_2_ breathing, are compared to those from a matched healthy group. Interactions were estimated by time series of successive beat-to-beat intervals (BBI), respiratory amplitude (RESP) at BBI onset, arterial systolic (SYS) and diastolic (DIA) blood pressures. The paper explores the Granger causality (GC) between systems in the frequency domain by the extended partial directed coherence (ePDC), considering instantaneous effects. Also, traditional linear and nonlinear markers as power in low (LF) and high frequency (HF) bands, symbolic dynamic indices as well as arterial baroreflex, were calculated. The results showed that for IPF during AA phase: 1) mean BBI and power of BBI-HF band, as well as mean respiratory frequency were significantly lower (*p* < 0.05) and higher (*p* < 0.001), respectively, indicating a strong sympathetic influence, and 2) the RESP → SYS interaction was characterized by Mayer waves and diminished RESP → BBI, i.e., decreased respiratory sinus arrhythmia. In contrast, during short-term SupplO_2_ phase: 1) oxygen might produce a negative influence on the systolic blood pressure variability, 2) the arterial baroreflex reduced significantly (*p* < 0.01) and 3) reduction of RSA reflected by RESP → BBI with simultaneous increase of Traube-Hering waves in RESP → SYS (*p* < 0.001), reflected increased sympathetic modulation to the vessels. The results gathered in this study may be helpful in the management of the administration of SupplO_2_.

## 1 Introduction

Idiopathic pulmonary fibrosis (IPF) is a chronic, restrictive, and progressive disease characterized by fibrosis and inflammatory changes in lung tissue. IPF also affects lung vasculature and prevents an adequate gas exchange, thus leading to exertional chronic arterial hypoxemia and dyspnea. The disease has a bad prognosis since after diagnosis the 50% of the patients die within 3–5 years (King et al., 2019). IPF etiology, diagnosis, treatment, and influences on quality of life, among other, have been investigated, but the focus has always been lungs performance. There is evidence that IPF patients also manifest comorbidities such as cardiovascular diseases, lung cancer, or pulmonary hypertension (Caminati et al., 2019). Furthermore, arterial hypertension has been related to dysfunctional autonomic cardiovascular control (Mancia and Grassi, 2014) and considerable impact on IPF disease progression and patient mortality (Buendía-Roldán et al., 2017).

Recently, IPF has been hypothesized as a systemic disease, but its influence on the autonomic nervous system (ANS) regulation has not been assessed as in other pulmonary disorders. For example, chronic obstructive pulmonary disease (COPD) negatively affects the cardiovascular system and the ANS regulation; the autonomic dysfunction is an important factor in the underlying pathophysiological mechanisms of the disease. The former conclusion has been mainly stated based on the analysis of heart rate variability (Van Gestel and Steier, 2010; Mohammed et al., 2015). However, for IPF disease studies about ANS regulation are scarce. Furthermore, supplemental oxygen (SupplO_2_) has been prescribed to IPF patients to improve clinical symptoms, but its impact on ANS regulation of cardiovascular and respiratory systems has not been evaluated in these patients.

The effects of oxygen supplementation have been reviewed on healthy subjects and patients with cardiovascular diseases, but on respiratory patients just for COPD. Until now the evidence about the benefits or drawbacks of oxygen supplementation is not conclusive. On the one hand, some authors point out that this type of clinical intervention does not help to increase oxygen delivery, i.e., the rate at which oxygen is transported from lung to microcirculation depending on cardiac output and arterial oxygen contents (Smit et al., 2018a). Furthermore, it is plausible that in critically ill patients, it could be associated with increased hospital mortality (You et al., 2018). Conversely, oxygen therapy in COPD patients is clinically well-accepted and it is recommended to enhance exercise capacity (Stoller et al., 2010). However, some trials have not found enough evidence to support that long-term oxygen therapy improves COPD patients’ mortality rate (Khor et al., 2019). It is important to point out that few studies have addressed the efficiency of oxygen administration in COPD and Interstitial Lung Disease (ILD) patients (Khor et al., 2019) and that studies about the ANS regulation in COPD patients were based on classic heart rate variability (HRV) parameters (Mohammed et al., 2017). Regarding IPF disorder, the authors of the present contribution compared the hemodynamic response to SupplO_2_ between ill and healthy subjects, showing potential detrimental effects of SupplO_2_ on IPF hemodynamics, particularly on total peripheral resistance (TPR) and cardiac output (Santiago-Fuentes et al., 2021).

Different methods have been employed to assess ANS regulation by the analysis of heart rate variability. In the case of HRV, it reflects the variations in the beat-to-beat interval and the corresponding variability time series is built up by diverse oscillatory modes. To extract the information from the time series, linear and nonlinear schemes have been proposed. For instance, the short-term HRV spectral density representation has been broadly used to analyze the sympathetic and parasympathetic modulation by the power in the low frequency (LF) and high frequency (HF) bands, respectively. Another way to assess ANS regulation of cardiovascular system is the nonlinear approach that allows to incorporate the analysis of complexity of underlying physiological mechanisms. Nonlinear indices by symbolic dynamics (SD) and detrended fluctuation analysis (DFA) have enabled the exploration of the cardiovascular system adaptability, among other, for example in elderly population (Beckers et al., 2006; Voss et al., 2015). Furthermore, the analysis of physiological variability time series has advanced from the univariate to bivariate and, to multivariate type to discover the complex interactions between human body subsystems. Nowadays, an open research area is to assess the cause-effect relationship to elucidate the complex picture of the autonomic control of the cardiovascular system and the complex interplay between cardiovascular and the respiratory systems.

Recently, the use of multivariate autoregressive models and the assessment of the directional interactions among a set of physiological variables (i.e., the so-called Granger causality) has been proposed for the analysis of ANS regulation under pathological and non-pathological conditions (Faes and Nollo, 2010; Faes et al., 2010; Charleston-Villalobos et al., 2019). Particularly, the extended partial directed coherence (ePDC) has gained interest as a tool to estimate the causality in the frequency domain in presence of instantaneous interactions (Faes and Nollo, 2010), i.e., as the effect from respiration to systolic blood pressure and beat to beat interval as well as the systolic blood pressure to beat to beat interval. Consequently, this study aimed to analysis cardiovascular and respiratory times series of variability by linear and nonlinear indices as well as Granger causalities (GC), via a multivariate autoregressive model including instantaneous effects, in IPF patients in comparison with healthy subjects under the effect of short-term SupplO_2_.

## 2 Materials and Methods

### 2.1 Subjects, Acquisition Protocol, and Preprocessing

**FIGURE 1:**
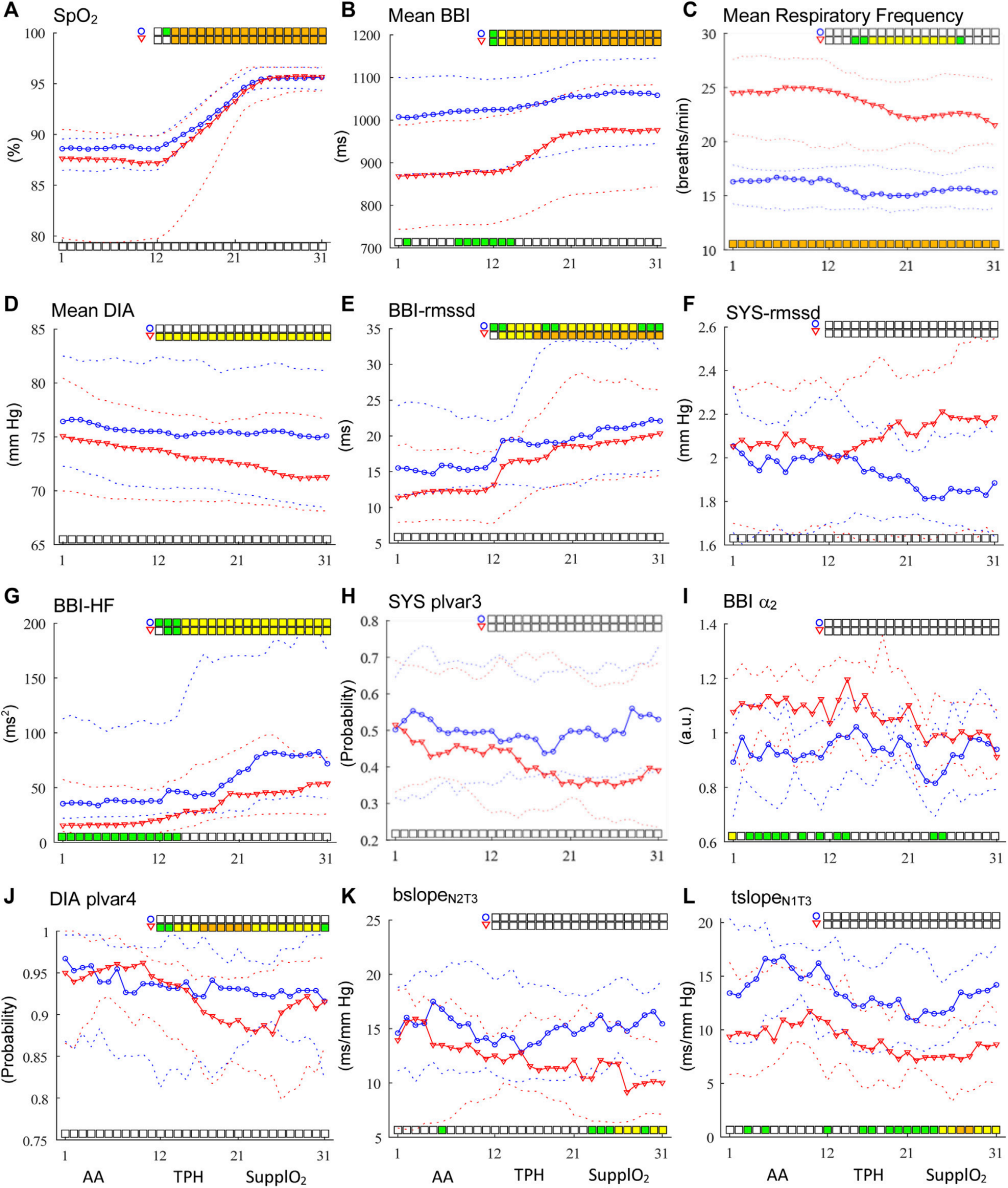
Time courses of linear and nonlinear indices for BBI, SYS and DIA time series **(A–L)**. AA phase occurs from windows 1 to 11, transition phase (TPH) from 12 to 20 while the steady SupplO_2_ from windows 21 to 31. Circles in blue represent healthy subjects and red triangles represent IPF patients. The solid line indicates the median values and broken lines indicate the interquartile range from 25 to 75%. Statistically significant differences between groups are shown with bars at the bottom of each graph while differences within-groups with bars at the top (*p* < 0.05 in green, *p* < 0.01 in yellow and *p* < 0.001 in orange).

This study includes 19 (8 women and 11 men) healthy subjects (CON) and 20 IPF patients (9 women and 11 men) with 67.79 ± 5.00 and 65.8 ± 6.48 years old, respectively. All subjects were medically evaluated at the National Institute of Respiratory Diseases in Mexico City after they accepted the invitation to participate and signed an informed consent according to the Declaration of Helsinki. Table 1 depicts different parameters related to clinical measures and respiratory functional tests of both groups. Signals acquisition was performed via a Biopac MP150 system during morning hours including ECG, continuous noninvasive arterial blood pressure, and peripheral blood oxygen saturation. Also, a thoracic belt was used to acquire the respiratory signal and all signals were sampled at 1,000 Hz. Raw signals were acquired in supine position continuously during 10 min with the subjects breathing spontaneously ambient air (AA) and an additional 10 min breathing SupplO_2_ at 3 L/min to ensure an arterial oxygen saturation above 94% (Santiago-Fuentes et al., 2021). Time series of successive beat-to-beat intervals (BBI), respiratory amplitude (RESP) at BBI onset as well as systolic (SYS) and diastolic (DIA) blood pressure (BP) were extracted from recorded signals; all extracted time series were manually reviewed and corrected. For GC analysis, the variability time series were resampled at 2 Hz using spline interpolation and normalized to zero mean and unit variance. For dynamic data analysis, consecutive windows of 5 min shifted by 30 s (90% overlap) were used. Therefore, the influence of SupplO_2_ on IPF was studied using 31 windows including three phases labelled as ambient-air (AA), transition phase (TPH) and steady supplemental oxygen (SupplO_2_), as indicated in Figure 1. The transition phase (TPH) is characterized by windows sharing AA and starting SupplO_2_ conditions. In each window, univariate, and bivariate indices, as well as the ePDC were estimated.

**TABLE 1:** Clinical and functional measures.

Measure	Control (19 W:8/M:11)	IPF (20 W:9/M:11)
Hematocrit (%)	47.4 ± 4.9	51.6 ± 2.4
Hemoglobin (g/dL)	15.3 ± 1.4	15.0 ± 1.4
Respiratory rate (bpm)	17 ± 4	25 ± 7*
Fibrotic HRCT scan	—	1.99 ± 0.53
FEV_1_ (%, predicted)	98.63 ± 12.87	78.15 ± 30.27*
FVC(%, predicted)	94.11 ± 12.72	72.40 ± 26.51*
FEV_1_/FVC	77.69 ± 6.37	88.08 ± 9.54*
D_LCO_ (%, predicted)	114.26 ± 20.85	67.20 ± 21.24*
PaO_2_(mmHg)	65.9^a^	61.92 ± 8.65
PaCO_2_(mmHg)	32.7^a^	34.76 ± 5.31

Values expressed as mean plus/minus standard deviation. FEV1, forced expiratory volume in one second; FVC, forced vital capacity; DLCO, diffusing capacity of the lungs for carbon monoxide; PaO2, partial pressure of oxygen in arterial blood; PaCO2, partial pressure of carbon dioxide.

a Estimated values for residents at the altitude of Mexico City.

* Statistical difference with *p*< 0.05.

### 2.2 Univariate and Bivariate Analyses

#### 2.2.1 Linear Analysis

Time and frequency domain linear indices were extracted as the mean value, the root mean square of successive differences (rmssd), and power in the very low-frequency range (VLF, 0.003–0.04 Hz), low (LF, 0.04–0.15 Hz), and high frequency (HF, frequencies >0.15 Hz) bands in agreement with the standardization proposed by the Task Force of the European Society of Cardiology and the North American Society of Pacing and Electrophysiology (AuthorAnonymous, 1996). The index rmssd is considered the more precise marker of chronotropic cardiac vagal influence (Minarini, 2020), for example, it provided a good differentiation between healthy subjects and vasovagal syncope patients for systolic and diastolic blood pressure variability (Reulecke et al., 2016). For estimating the power of LF and HF bands a parametric autoregressive model was used in each temporal window along of the variability time series. The model parameters and optimal order were estimated using the Burg method and the Akaike Information Criterion (AIC), respectively.

#### 2.2.2 Nonlinear Analysis

To explore the nonlinear time series properties two techniques were applied: Detrended Fluctuation Analysis (DFA) to quantify the fractal scaling properties of a time series, and Symbolic Dynamics (SD), which is a coarse-grained method based on symbols (Reulecke et al., 2016). For DFA analysis, the short-term fractal scaling exponent (α_1_) was calculated over equal and non-overlapping segments with length between 4 and 16 beats while the long-term exponent (α_2_) for segments with length of 16–64 beats (Peng et al., 1995). Furthermore, by SD technique, nonlinear indices related to the low or high variability of the signal were determined. An alphabet, consisting only of symbols “0” or “1”, was used to create words of length six and for BBI time series, thresholds of 2, 5, 10 and 20 ms were established (Voss et al., 2015). In this sense**,** a low variability index associated with the word “000000” and a high variability index associated to the word “111111”, were counted (Schulz and Voss, 2017). In the case of blood pressure variability (BPV), the 6-length words were created by selecting thresholds of 1, 2, 3 and 4 mmHg (plvar 3 and 4) (Reulecke et al., 2016).

#### 2.2.3 Bivariate Analysis

The Dual Sequence Method (DSM) is a linear technique to estimate the arterial baroreflex sensitivity (BRS) by the analysis of spontaneous fluctuations in systolic BP and BBI time series. Traditionally, a pattern of three consecutive increments or decrements in SYS and BBI are tagged as bradycardic (bslope, increase in SYS that causes an increase in BBI) or tachycardic (tslope, a decrease in SYS that causes a decrease in BBI) sequences, respectively. However, we avoided using the classical thresholds for SYS and BBI due to the criticisms about them (Gouveia et al., 2007) and in consequence, different pattern length was used including one up to three samples (N1-N3). Furthermore, the synchronous responses in the same beat interval (T0) and delayed BBI responses shifted by one up to three beats (T1-T3) were also explored, as suggested by (Malberg et al., 2002; Gouveia et al., 2007).

### 2.3 Multivariate Autoregressive Modeling, Extended Partial Coherence and Time-Frequency Analysis of Interactions

Establishing the cause and effect, or the driver-response relationship, between physiological systems has been of great interest in diverse biomedical applications. Fundamental to the driver-response relationship is the concept of Granger causality which states that if a signal improves the prediction of a second signal, above and beyond, its prediction in terms of its own past, then the first signal causes the second one. GC has been formalized in terms of multivariate time series analysis and its treatment in the frequency domain leading to the concept of partial directed coherence. Particularly, the extended partial directed coherence (ePDC) is based on fitting a linear time-invariant parametric model to the observed set of *M* time series ***Y*** (*n*) including instantaneous effects, i.e.,

$$Y\left(n\right)=\overset{q}{\underset{k=0}{\sum }}B\left(k\right)Y\left(n-k\right)+W\left(n\right),$$ (1)

where the model coefficients ***B*** (*k*), *k* = 0, … ,*q*, are related to instantaneous and strictly causal effects, *q* is the model order and ***W*** (*n*) is the innovation process formed by white and uncorrelated noises with diagonal covariance matrix **Λ** = diag (λ_i_^2^). The identification of the extended MVAR model can be achieved from a strictly causal MVAR model with coefficient matrix $\hat{A}\left(k\right),\;k=1,\ldots .,q,$ as $\hat{B}\left(k\right)=\left[I-B\left(0\right)\right]\hat{A}\left(k\right)$ (Faes et al., 2010; Charleston-Villalobos et al., 2019). Furthermore, to calculate ***B*** (0) is necessary to perform a Cholesky decomposition of the input covariance matrix $\text{Σ}=L\text{Λ}L^{T}$ , with Σ=cov(U(n)) , of the strictly causal model to obtain the matrix $L={\left(I-B\left(0\right)\right)}^{-1}$ . From this decomposition ***L*** is a lower triangular matrix as well as ***B*** (0) with null diagonal, but it is required to order the times series in ***Y*** (*n*) to set the direction, but not the strength, of physiological influence among them. Consequently, in the present study, to achieve the former constrain, two models were proposed: i) MVAR model 1 (MVAR_1_) where ***Y*** (*n*) was built up as *y*_1_ = RESP, *y*_2_ = SYS and *y*_3_ = BBI and ii) MVAR model 2 (MVAR_2_) with *y*_1_ = RESP, *y*_2_ = DIA, and *y*_3_ = BBI. The model order *q* was obtained by the minimum of the function $AIC\left(q\right)=Nlog\left(det\Sigma \right)+M^{2}q$ , where **Σ** is the input covariance matrix of the strictly causal model. From the frequency domain representation of Eq. 1 is possible to obtain the spectral density of ***Y*** (*f*) and its inverse, from which the partial coherence (PC) function Π_*ij*_(*f*) between *y*_*i*_ and *y*_*j*_ is defined. However, PC cannot provide information about causality due to its symmetrical nature and, consequently, a factorization of Π_*ij*_(*f*) is necessary to produce ePDC, named $\chi_{ij}\left(f\right)$ , as:

$$\chi_{ij}\left(f\right)=\frac{\left(\frac{1}{\lambda_{i}}\right){\bar{B}}_{ij}\left(f\right)}{\sqrt{\sum_{m=1}^{M}\left(1/\lambda_{m}^{2}\right){\left|{\bar{B}}_{mj}\left(f\right)\right|}^{2}}}.$$ (2)

where ${\bar{B}}_{ij}\left(f\right)=1-\overset{q}{\underset{k=0}{\sum }}B_{ij}\left(k\right)e^{-2\pi fkT}$ with *T* equal to the sampling period and λ_*i*_ is a diagonal value of the noise covariance matrix of the ***W*** process in Eq. 1. Therefore, ePDC is a directional frequency domain measure of connectivity quantifying the influence of the process *y*_*j*_ on the process *y*_*i*_, removing the influence of other processes. The ePDC is normalized with respect to the structure that sends the signal taking values between 0 (absence of causal coupling) and 1 (full causal coupling) at frequency *f*. In this study, the identification of eMVAR model was performed by the standard least-squares method and the estimated ePDC was corrected by statistical hypothesis testing based on setting a threshold for significance using causal Fourier transform surrogates (Faes et al., 2010; Charleston-Villalobos et al., 2019). In a few words, once the eMVAR model is estimated, if there is a direct causality from the process $y_{j}$ to $y_{i},$ the corresponding coefficients $b_{ij}\left(k,w_{l}\right),\;k=0,\ldots ,q,$ at each temporal window (*w*_*l*_) are set to zero, and from there the surrogates are obtained; causality is selectively destroyed only over the direction under study, details of the procedure can be found in Faes et al., 2010. To accept or reject the estimated ePDC, its magnitude at each frequency was compared with the threshold obtained from 100 surrogates (95% confidence interval). If the magnitude of ePDC was below the threshold, the null hypothesis was accepted, reflecting the absence of interaction, and the estimated value was set to zero. In the present study, the time series of variability were segmented producing a time-frequency representation of interactions (TFR_i_). In TFRi, the x-axis is related to the 31 temporal windows of 5 min and the y-axis depicts the frequency in Hz (0.0–0.50 Hz) using 512 bins. Furthermore, the z-axis represents the magnitude of the ePDC (0–0.4) by a color palette from blue to red, respectively. To count with the same number of samples in each window a resampling was performed at 2 Hz. It is worthy to note that, at least for this research protocol, the TFRi without or with resampling are close to each other and the dynamic changes produced by IPF, and supplemental oxygen remain.

### 2.4 Statistical Analysis

To evaluate the effect of supplemental oxygen, in this study the statistical analysis was performed with two statistical test, within-groups and between-groups. The within-groups test was carried out on univariate and bivariate indices values comparing window 5 vs. windows 12 to 31 (TPH and steady SupplO_2_) by the Wilcoxon Sign Rank-sum. All *p*-values were corrected using the Benjamini and Hochberg correction (Benjamini and Hochberg, 1995). In the case of between-groups comparison, the analysis was carried out on the univariate and bivariate indices values, and ePDC magnitude at each tile, in each temporal window by the nonparametric Mann-Whitney-U-test. In both statistical analyses, the significance was set at three levels for descriptive purposes: slightly significant for *p* < 0.05 (green), moderately significant for *p* < 0.01 (yellow), and highly significant for *p* < 0.001 (orange).

## 3 Results and Discussion

The section is organized presenting first the results for the AA phase followed by the findings in the TPH and steady SupplO_2_ phases. The SupplO_2_ effect on healthy subjects and IPF patients is obtained looking for the time course of linear and nonlinear indices as well as by the dynamics of cardiovascular and respiratory interactions as compared with those in the AA phase. Statistical analysis between-groups, throughout all the phases, is displayed at the bottom of the graphs while within-group, comparing window 5 vs. windows 12 (start of TPH) to 31, is depicted at the top. For saving the space of article, selected indices are included in Figure 1, while others are only discussed. Also, the indices in Figure 1 were ordered as they are used throughout the manuscript.

### 3.1 Clinical and Functional Measurements

According to Table 1, the IPF group was characterized by moderate reduction in vital capacity and FEV1, mild reduction in diffusion capacity, hypoxemia, mild hypercapnia, and high respiratory rate. In contrast, the CON group had no signs of pulmonary alterations; although the PaCO_2_ and PaO_2_ were not registered at the time of the study, it is plausible to estimate them at the altitude of Mexico City (Vazquez-Garcia and Perez-Padilla, 2000). Consequently, the estimated normal values of PaCO_2_ and PaO_2_ for Mexico City residents are around 32.7 and 65.9 mmHg, respectively; there were no statistically significant differences in age, anthropometric measures, hematocrit, and hemoglobin values. As can be seen in Figure 1A, peripheral oxygen saturation (SpO_2_) increased importantly from TPH towards the steady SupplO_2_ phase for both groups until a saturation of 96% was reached, but no statistically significant differences between-groups were found during SupplO_2_. In contrast, within-group statistical analysis revealed highly significant differences. Furthermore, Rico et al. observed that S_P_O_2_ tends to decline during the aging process that is in line with the SpO_2_ in our Control group (Rico et al., 2001). On the other hand, the IPF group showed a greater dispersion of SpO_2_ during AA phase that may be explained by age, disease stage, and pulmonary condition. Also, it is relevant to point out that the population of this age was under medication for other comorbidities. A comprehensive discussion of this aspect for the groups under study can be found in (Santiago-Fuentes et al., 2021).

### 3.2 Linear and Nonlinear Univariate Analysis in AA

For mean BBI index, statistically significant differences between CON and IPF groups were found from windows 8 to 12, Figure 1B, while for the mean respiratory frequency highly significant differences were found for the whole phase, Figure 1C. Also, the mean SYS and DIA BP for CON tended to be higher than for IPF, Figure 1D. For linear indices as rmssd, there were no significant differences for BBI, SYS or DIA, Figures 1E,F, but for BBI the IPF group showed a tendency to lower values. Also, for BBI power in the LF and HF bands was significantly different (*p* < 0.05); particularly, the CON group showed higher BBI-HF power and consequently, higher cardiac vagal influence than IPF, Figure 1G. For SYS-nLF, IPF showed a tendency to higher values, i.e., increased sympathetic influence on the vasculature than in CON. In the case of SD nonlinear analysis, for IPF the indices BBI-phvar2 as well as SYS-plvar3 (Figure 1H) showed a tendency to lower probability values than CON, i.e., low BBI variability and higher SYS variability, respectively**.** Nevertheless, in the DFA analysis, BBI-α_2_ was statistically different (*p* < 0.05) between groups, where IPF patients showed higher sympathetic activity than CON, Figure 1I. Consequently, by univariate analysis, statistically significant differences between groups were found just for BBI, indicating a significant cardiac sympathetic modulation in IPF patients during AA scenario.

### 3.3 Linear and Nonlinear Univariate Analysis in TPH and Steady SupplO_2_

At the firsts windows in TPH, BBI showed statistically significant differences between-groups for mean, BBI-HF, and BBI-α_2_ indices, Figure 1. Specifically, the mean cardiac frequency decreased in both groups, slowly for CON and more drastically for IPF, whereas for HF and α_2_, the CON group showed higher and lower values than IPF, respectively. The former behavior is in line with the expected effect of SupplO_2_ on the cardiac frequency. The mean respiratory frequency was reduced for both groups however, the between groups differences were kept along both phases, Figure 1C. Although between-groups no statistically significant differences were found for mean SYS and DIA in TPH or steady SupplO_2_, the mean SYS showed a tendency to increase in CON while the mean DIA decreased in IPF.

To evaluate the influence of O_2_ within each group, statistical comparisons were achieved between AA and TPH or steady SupplO_2_ phases. The effect of O_2_ was significantly different for CON and IPF. Specifically, significant differences were obtained for BBI, mean respiratory frequency, and DIA. For the IPF group, the BBI-rmssd index showed highly significant differences (*p* < 0.001) from windows 16 to 31, i.e., the cardiac vagal influence in IPF was increased to a greater extent by O_2_, Figure 1E. The former time course was supported by the nonlinear BBI-phvar2 index (*p* < 0.001). Furthermore, BBI-LF and BBI-α_1_ indices provided significant within-group differences. Regarding BBI-LF index, only for the IPF group, the LF power increased from TPH and throughout steady SupplO_2_ phase (*p* < 0.001) while the BBI-α_1_ index decreased. It is worthy to note that for SYS, in none of the groups neither linear nor nonlinear indices provided significant differences. Although, for IPF, SYS-rmssd had a tendency to increase while SYS-plvar3 to decrease, indicating that SYS increased its variability with O_2_, Figures 1F,H. In the case of DIA, just for IPF, mean DIA decreased due to oxygen from TPH to the end of the steady SupplO_2_ phase in a moderately significant way (*p* < 0.01), that may be related to the lower cardiac frequency with O_2_, i.e., a vagal modulation effect, Figure 1D. Also, the DIA-plvar4 index showed moderately and highly significant differences along the two phases, the percentage of words of low variability was reduced, i.e., in the IPF group the DIA variability was increased by SuppO_2_, Figure 1J.

### 3.4 Bivariate Analysis in AA, TPH and Steady SupplO_2_

For DSM analysis, different pattern lengths and delays were tested, and the results indicated that statistical differences between groups occurred with lengths of 2–3 samples and shifts between 0 and 3 beats. The bivariate indices during AA, associated with arterial baroreflex sensitivity, did not show statistical differences between groups, only a tendency to lower values for IPF. The former behavior is in line with the literature indicating that hypoxia produces a resetting of arterial baroreflex, without changing the sensitivity, to higher heart rates and systolic blood pressures due to stimulation of peripheral chemoreceptors (Halliwill et al., 2003). In contrast, the IPF patients of the present study were characterized by significantly higher cardiac frequency and cardiac sympathetic modulation but similar systolic pressure. It is worthy to mention that the interaction between baroreflex and peripheral chemoreflexes remains controversial (Kronsbein et al., 2020). During steady SupplO_2_, from window 21, bslope and tslope indices provided significant differences (*p* < 0.05 and *p* < 0.01) between groups, Figures 1K, 1L. For both indices, IPF showed lower values than CON, i.e., IPF decreased their baroreflex in comparison to CON. In the case of within-groups analysis, in TPH and steady SupplO_2_ phases, bslope tends to increase for CON while for the IPF group tends to decrease. Systolic pressure, being a variable to control, tends to oscillate with greater amplitude when feedback mechanisms are deficient, showing an inverse relationship with variations in heart rate, as a variable to regulate (Mancia et al., 1986; Lanfranchi and Somers, 2002). In fact, it should be noted that the significant reduction in cardiac output in IPF observed during SupplO_2_ (Santiago-Fuentes et al., 2021) could be a consequence of alteration of the cardiovascular regulation. The former behavior may be explained, on the one hand, by statistically significant changes (attenuation) of the sensitivity of the baroreceptors reflected by bslope and tslope indices (Figures 1K,L), increase in BBI (Figure 1B), and in total peripheral resistance and, on the other hand, by statistically non-significant trends in systolic blood pressure and its variability. Furthermore, diverse research pointed out that clinicians should be aware of the prognostic implications of increased blood pressure variability, a marker of cardiovascular decompensation, which may lead for example to organ damage (Hoecht, 2013).

### 3.5 Interactions Between Cardiovascular and Respiratory Systems

#### 3.5.1 TFR_i_ Magnitude Distribution in AA

**FIGURE 2:**
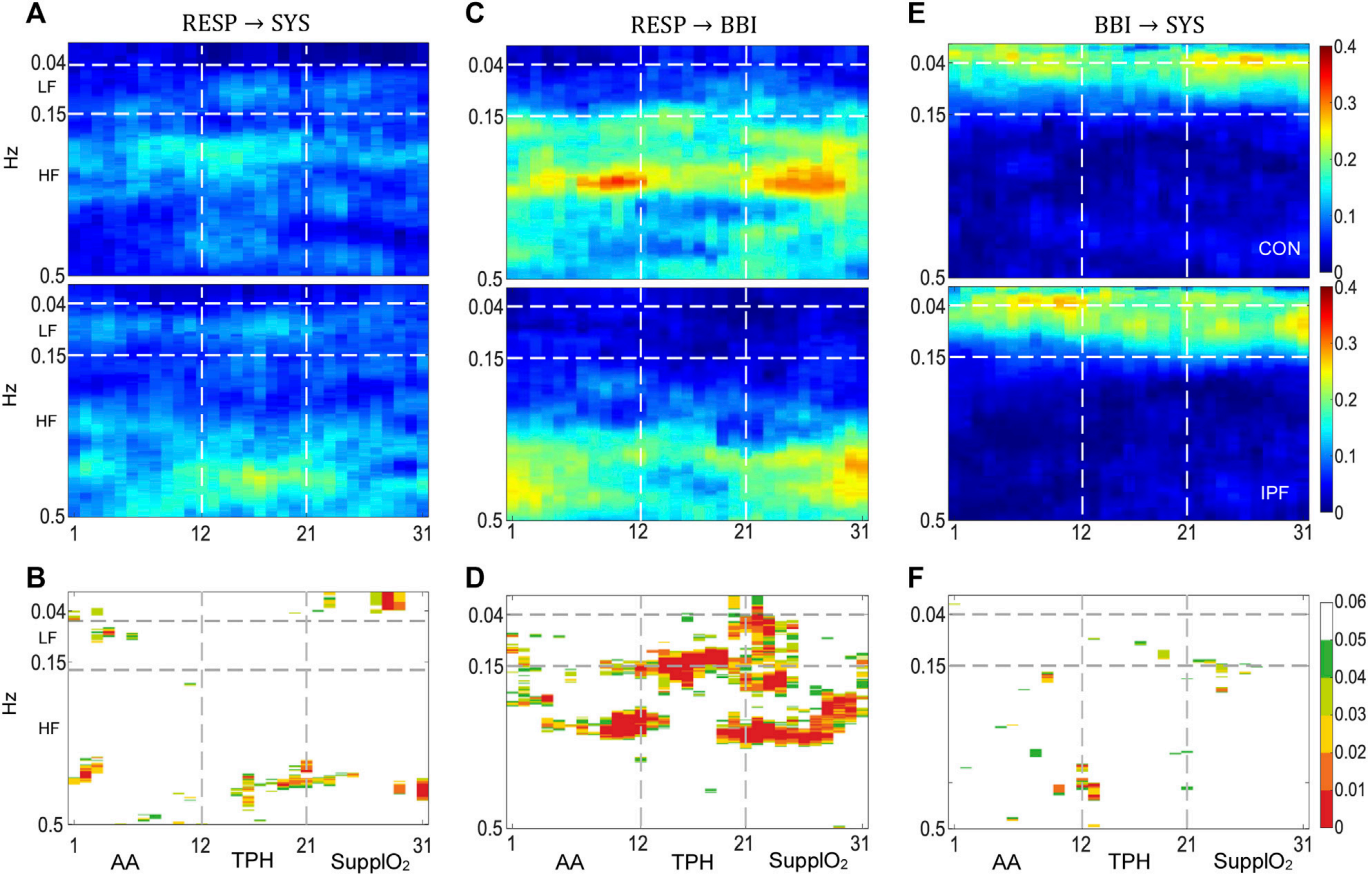
Cadiorespiratory and cardiovascular interactions by the model MVAR_1_. **(A,C,E)** TFR_i_ of RESP → SYS, RESP → BBI and BBI → SYS interactions, respectively, **(B,D,F)** the map of corresponding statistical differences between groups [*p* < 0.05 (green), *p* < 0.03 (yellow), *p* < 0.01 (red)]. For each interaction, the TFRi of the control group is at the top while the one for patients is at the bottom of the panel. Horizontal broken lines indicate the LF and HF bands while the vertical lines mark the phases of the protocol.

An averaged time-frequency representation of RESP → SYS interaction by MVAR_1_, associated with the information flow from RESP to SYS, is showed in Figure 2A, for CON (above) and IPF group (below). The TFR_i_ magnitude distribution points out differences between groups for the LF and HF bands, the significant differences are displayed in Figure 2B. The power in the LF band has been associated with the sympathetically mediated BP vasomotor modulation, the so-called Mayer waves around 0.1 Hz. The origin of Mayer waves has not been elucidated, but some authors agreed that these waves could be related to an oscillatory sympathetic activation that, in the specific case of humans, is independent of factors such as gender, age, or posture and can be induced in BP by hypoxia (Julien, 2006; Ghali and Ghali1, 2020). According to the RESP → SYS interaction in the LF band, the TFRi magnitude for IPF is higher than for CON that is in line with the SYS univariate results presented above; the IPF group showed a tendency to an augmented sympathetic activity in AA, as revealed by SYS-rmssd (Figure 1F). Furthermore, for the CON group the magnitude of the TFR_i_ in the HF band is concentrated around 0.28 Hz while for IPF group is spread out around 0.42 Hz, i.e., around the respective mean respiratory frequency, Figure 1C. It is plausible that different mechanical effect on central blood volumes is produced by higher respiratory frequency and lower FVC in IPF than in CON. Also, in the HF band Traube-Hering waves (THW) have been reported associated with respiratory-related fluctuations in sympathetic outflow that promotes changes in vascular tone (Menuet et al., 2020). In fact, the IPF group showed a significant increased TPR during AA, i.e., increased vascular tone (Santiago-Fuentes et al., 2021). In the case of SYS → BBI interaction there were no statistically significant differences and consequently, the corresponding TFR_i_ is not shown. A possible explanation could be associated to the fact that subjects were in supine position and then, baroreflex feedback control may be blunted. The RESP → BBI interaction, Figure 2C, shows that the magnitude for CON is spread out over the whole HF band and is higher than for IPF that is localized around the mean respiratory frequency, the corresponding statistical differences are displayed in Figure 2D. Therefore, the magnitude of the RESP → BBI interaction pointed out that respiratory sinus arrhythmia (RSA), associated with the cardiac vagal influence, occurs at different operating point in CON and IPF. The former result is in line with other studies which suggest that RSA decreases with hypoxia (Yasuma et al., 2001) and in fact, the IPF group of the present study was characterized by hypoxemia (Table 1). Furthermore, RSA was more relevant for CON as the magnitude of the RESP → BBI interaction is higher than the corresponding of the RESP → SYS interaction, i.e., the influence of RESP on the heart is higher than on the vascular subsystem. Regarding the BBI → SYS interaction, Figure 2E, associated with the mechanical feedforward influence, a relevant magnitude is located towards the VLF and LF bands for both groups; however, there were no statistically significant differences.

**FIGURE 3:**
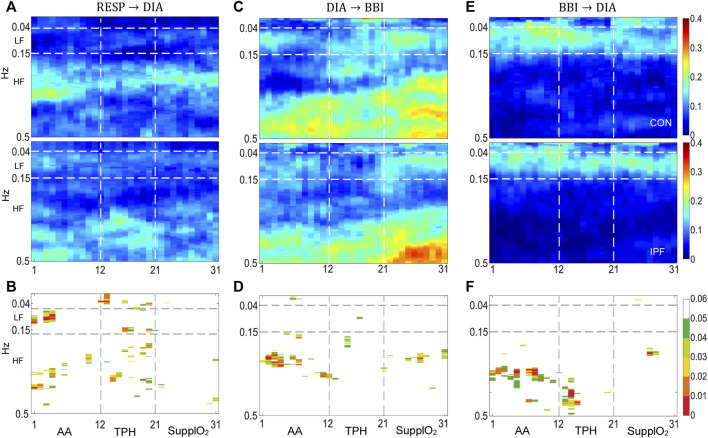
Cadiorespiratory and cardiovascular interactions by the model MVAR_2_. **(A,C,E)** TFR_i_ of RESP → DIA, DIA → BBI and BBI → DIA interactions, respectively, **(B,D,F)** the map of corresponding statistical differences between groups [*p* < 0.05 (green), *p* < 0.03 (yellow), *p* < 0.01 (red)]. For each interaction, the TFRi of the control group is at the top while the one for IPF patients is at the bottom of the panel. Horizontal broken lines indicate the LF and HF bands while the vertical lines mark the phases of the protocol.

Results by MVAR_2_, using DIA instead of SYS are shown in Figure 3. The TFR_i_ of the RESP → DIA interaction is shown in Figure 3A and resembles the TFR_i_ of RESP → SYS, but for both groups the magnitude spread further in the LF and HF bands, statistically significant differences are shown in Figure 3B. For the DIA → BBI interaction, Figure 3C, the TFRi displayed a magnitude with a trend to be higher in CON, particularly in the upper part of the HF band, while for the IPF group a more uniform distributed magnitude is observed across the frequency bands. Furthermore, for both groups the DIA → BBI interaction presents a higher magnitude than SYS → BBI interaction. The former behavior may be interpreted in terms of an impaired left ventricular (LV) diastolic filling in IPF in contrast to a preserved LV systolic function, as was shown in a previous study (Papadopoulos et al., 2008). Also, the lower magnitude in the DIA → BBI interaction for the IPF group may reflect peripheral vascular changes due to increased arterial stiffness and in conjunction with a high heart rate may be indicative of the prevalence of sympathetic tone. For the BBI → DIA interaction, the TFR_i_ magnitude distribution resembles the corresponding to BBI → SYS of MVAR_1_, Figure 3E, but with a tendency to display lower values for both groups. Furthermore, a consistent activity in the LF band was found, the Mayer wave. For the DIA → RESP interaction, the corresponding TFR_i_ did not show relevant magnitude in any phase and, consequently it was not included in the paper. Therefore during the AA phase, based on the TFR_i_ magnitude, its spread and relevance of statistically significant differences between groups, IPF during the AA phase mainly impacted the RESP → SYS and RESP → BBI interactions.

#### 3.5.2 TFR_i_ Magnitude Distribution in TPH and Steady SupplO_2_

In the case of RESP → SYS interaction, during TPH the SupplO_2_ increased the TFR_i_ magnitude in both groups, Figure 2A. For the CON group the magnitude spread further in the LF and HF (the Traube-Hering waves) bands as compared with the AA phase. In contrast, for the IPF group the magnitude was increased mainly in the upper part of the HF band, the statistically significant differences are shown in Figure 2B. Furthermore, in the steady SupplO_2_ phase, the TFR_i_ magnitude decreased in both groups but remains higher in IPF, it seems that the interaction for both groups reached an operation level like the level in AA phase. Presumably, in IPF the heightened arterial peripheral chemoreceptors activity/sensitivity by the hypoxia keeps the drive of RESP on SYS (Stickland et al., 2016). In fact, chronic hypoxia produces among other things, hyperplasia of the carotid body and increases its activity. As can be seen in Figure 1C, throughout the SupplO_2_ phase the mean respiratory frequency decreased in the IPF group, with a moderate *p*-value with respect to AA, but the influence on SYS does not change significantly.

During TPH, the RESP → BBI interaction reveals that RSA was highly significant different between CON and IPF groups, Figures 2C,D, i.e., RSA in CON was stronger. The former result is in line with the fact that vagal modulation facilitates RSA at rest while sympathetic activation attenuates its magnitude. Moreover, for the RSA physiological purpose, there are three major hypotheses, i.e., 1) improvement of gas exchange, 2) minimization of the energy consumption for the heart, and 3) reduction of BP fluctuations (Buchner, 2019). One of the premises of hypothesis 3) is that suppression of RSA generally increases BP fluctuations in its HF band. In fact, the former hypothesis may explain the decrease in RSA for the IPF group that may be related to the tendency to increase of SYS-rmssd and SYS-phvar1 indices, through SupplO_2_ as discussed above. During the steady SupplO_2_ phase the RESP → BBI interaction showed highly significant differences. For CON, the TFR_i_ significant magnitude in the HF band, almost in the whole phase, reveals that SupplO_2_ affects importantly respiratory drive on BBI. In contrast, for the IPF group the RESP → BBI interaction remains at similar operation level as previous phases, in consequence, the RSA in IPF was lower than in CON. It is noteworthy that in SupplO_2_ phase, for the CON group the RESP → BBI (RSA) interaction increased while RESP → SYS magnitude (THW) decreased (Barnett et al., 2020). In the case of BBI → SYS, for IPF during TPH, the TFR_i_ magnitude showed a sustained level of driving indicating a higher influence of the cardiac mechanical modulation, Figure 2E. In steady SupplO_2_, in the LF band there were no significant differences, but there was a trend in IPF towards a greater magnitude, which is associated with sympathetic modulation on the vasculature.

By MVAR_2_ using DIA instead of SYS, the RESP → DIA interaction reveals that the TFRi magnitude for CON and IPF is concentrated around the mean respiratory frequency of each group as occurs for RESP → SYS, Figures 3A,B. It seems that the oxygen delivery did not influence significantly the information flow from RESP to DIA. Regarding the DIA → BBI interaction, Figure 3C, the TFR_i_ magnitude in steady SupplO_2_ phase showed some differences between groups in the HF band, and mainly toward the end of the phase, statistically significant differences are depicted in Figure 3D. A plausible explanation is that the diastolic BP is related to the pumping activity of the aorta and large arteries of the circulating system. So factors such as systemic vascular resistance or arterial stiffness modify diastolic BP more than systolic BP, and therefore, the detriment of baroreflex activity at rest, as reflected by the tslope index in Figure 1L, is more evident in DIA → BBI than in SYS → BBI. In the case of the BBI → DIA interaction, Figure 3E, the TFR_i_ resembles the behavior of BBI → SYS interaction in the LF band and it seems to be slightly affected by oxygen but there were no statistically significant differences, Figure 3F. It is worthy to note that stationarity is assumed for temporal windows of 5 minutes of cardiovascular and respiratory time-series. However, during TPH nonstationary behavior may be elicited and consequently, a modified time-frequency approach could be applied.

## 4 Conclusion

According to the results of the present study, the IPF group in the AA phase, as compared with a matched healthy group, was characterized by high sympathetic modulation to the heart (supported by BBI-rmssd, BBI-α_2_ indices, and increased mean respiratory frequency, among other indices) with a decreased influence of the parasympathetic activity. It is well-known that chemoreceptors sense the partial pressure of oxygen in blood vessels, and they are important modulators of sympathetic activation in response to hypoxemia. The activation is also known as chemoreflex-mediated sympathetic activation and one of the consequences is the hyperventilation that leads to the inhibition of the chemoreflex due to the stretch mechanoreceptors activity in the thoracic cage (Kara et al., 2003). Also, the IPF patients during SupplO_2_ had blunted baroreflex (confirmed by DSM results and almost inexistent SYS → BBI interaction), as well as RSA activity (confirmed by BBI-HF and RESP → BBI interaction). The former autonomic behavior could be explained partially by the chronic hypoxemia and hypercapnia in the IPF group. In this study, the patient group suffered mild hypoxia and some evidence of hypercapnia, so the enhanced sympathetic activity and reduced parasympathetic response may be a consequence of alterations in baroreceptors and peripheral chemoreceptors, which reduce the response to supplemental oxygen. In fact, Van Gestel and Steier showed evidence related to the high sympathetic activity in COPD patients due to the impaired baroreflex sensitivity altered by hypoxia but not by hypercapnia (Van Gestel and Steier, 2010). Additionally, an impairment of cardiac control and sympathetic overactivity may be related to age, but these responses seem to be higher for the IPF group. In fact, Porta et al. at supine rest phase, found a possible age-related impairment of the cardiac control and altered response to stressors in conjunction with a gradual decrease in SYS complexity and thus, an increase of the sympathetic activity (Porta et al., 2014).

The effect of oxygen has been studied mainly by its hemodynamic effects and the analysis of heart rate variability (Lund et al., 1999; Gole et al., 2011; Smit et al., 2018a; Smit et al., 2018b). To the best of our knowledge, this is the first study that tackle the effect of oxygen supplementation in IPF with a new perspective from the point of view of linear and non-linear indices as well as the dynamics of cardiovascular and respiratory systems interactions. For IPF patients the results showed that during AA phase: 1) the mean BBI value and power of BBI-HF band, as well as the mean respiratory frequency were significantly lower (*p* < 0.05) and higher (*p* < 0.001), respectively, indicating a strong sympathetic influence, and 2) the RESP → SYS interaction was characterized by Mayer waves and diminished RESP → BBI, i.e., decreased respiratory sinus arrhythmia. In contrast, for IPF during short-term SupplO_2_ phase: 1) oxygen might produce a negative influence on the systolic blood pressure variability (SYS-rmssd was increased among other indices), 2) the arterial baroreflex reduced significantly (*p* < 0.01), and 3) reduction of RSA (RESP → BBI) with simultaneous increase of Traube-Hering waves (RESP → SYS), reflected increased sympathetic modulation to the vessels. Our study in patients with IPF, compared with control subjects residing at the same altitude level, suggests that the autonomic alterations induced by the pathology persist or worsen despite the acute administration of oxygen. Based on our previous effort, indicating a relevant increase of TPR (Santiago-Fuentes et al., 2021), current research by the group is directed to analyze interactions including TPR. Finally, the proposed TFRi analysis may be used to better understanding the underlying physiological phenomena of different respiratory diseases.

## Data Availability

The original contributions presented in the study are included in the article/Supplementary Material, further inquiries can be directed to the corresponding author.

## References

[B1] AuthorAnonymous (1996). Heart Rate Variability: Standards of Measurement, Physiological Interpretation and Clinical Use. Task Force of the European Society of Cardiology and the North American Society of Pacing and Electrophysiology. Circulation 93 (5), 1043–1065. 8598068

[B2] Barnett W. H. Latash E. M. Capps R. A. Dick T. E. Wehrwein E. A. Molkov Y. I. (2020). Traube-Hering Waves Are Formed by Interaction of Respiratory Sinus Arrhythmia and Pulse Pressure Modulation in Healthy Men. J. Appl. Physiol. 129 (5), 1193–1202. 10.1152/japplphysiol.00452.2020 32940558 PMC7790131

[B3] Beckers F. Verheyden B. Aubert A. E. (2006). Aging and Nonlinear Heart Rate Control in a Healthy Population. Am. J. Physiology-Heart Circulatory Physiol. 290 (6), H2560–H2570. 10.1152/ajpheart.00903.2005 16373585

[B4] Benjamini Y. Hochberg Y. (1995). Controlling the False Discovery Rate: A Practical and Powerful Approach to Multiple Testing. J. R. Stat. Soc. Ser. B (Methodological) 57 (1), 289–300. 10.1111/j.2517-6161.1995.tb02031.x

[B5] Buchner T. (2019). A Quantitative Model of Relation between Respiratory-Related Blood Pressure Fluctuations and the Respiratory Sinus Arrhythmia. Med. Biol. Eng. Comput. 57 (5), 1069–1078. 10.1007/s11517-018-1939-4 30578447 PMC6476852

[B6] Buendía-Roldán I. Mejía M. Navarro C. Selman M. (2017). Idiopathic Pulmonary Fibrosis: Clinical Behavior and Aging Associated Comorbidities. Respir. Med. 129, 46–52. 10.1016/j.rmed.2017.06.001 28732835

[B7] Caminati A. Lonati C. Cassandro R. Elia D. Pelosi G. Torre O. (2019). Comorbidities in Idiopathic Pulmonary Fibrosis: an Underestimated Issue. Eur. Respir. Rev. 28 (153), 190044. 10.1183/16000617.0044-2019 31578211 PMC9488913

[B8] Charleston-Villalobos S. Reulecke S. Voss A. Azimi-Sadjadi M. R. González-Camarena R. Gaitán-González M. J. (2019). Time-Frequency Analysis of Cardiovascular and Cardiorespiratory Interactions during Orthostatic Stress by Extended Partial Directed Coherence. Entropy 21 (5), 468–482. 10.3390/e21050468 33267182 PMC7514957

[B9] Faes L. Nollo G. (2010). Extended Causal Modeling to Assess Partial Directed Coherence in Multiple Time Series with Significant Instantaneous Interactions. Biol. Cybern 103 (5), 387–400. 10.1007/s00422-010-0406-6 20938676

[B10] Faes L. Porta A. Nollo G. (2010). Testing Frequency-Domain Causality in Multivariate Time Series. IEEE Trans. Biomed. Eng. 57, 1897–1906. 10.1109/TBME.2010.2042715 20176533

[B11] Ghali M. G. Z. Ghali G. Z. (2020). Mechanisms Contributing to the Generation of Mayer Waves. Front. Neurosci. 14, 395. 10.3389/fnins.2020.00395 32765203 PMC7381285

[B12] Gole Y. Gargne O. Coulange M. Steinberg J.-G. Bouhaddi M. Jammes Y. (2011). Hyperoxia-induced Alterations in Cardiovascular Function and Autonomic Control during Return to Normoxic Breathing. Eur. J. Appl. Physiol. 111 (6), 937–946. 10.1007/s00421-010-1711-4 21069379

[B13] Gouveia S. Rocha A. P. Laguna P. Lago P. (2007). Threshold Sensitivity in Time Domain BRS Estimation: Minimum Beat-To-Beat Changes and Minimum Correlation. Comput. Cardiol. 34, 557–560. 10.1109/CIC.2007.4745546

[B14] Halliwill J. R. Morgan B. J. Charkoudian N. (2003). Peripheral Chemoreflex and Baroreflex Interactions in Cardiovascular Regulation in Humans. J. Physiol. 552 (1), 295–302. 10.1113/jphysiol.2003.050708 12897165 PMC2343329

[B15] Höcht C. (2013). Blood Pressure Variability: Prognostic Value and Therapeutic Implications. ISRN Hypertens. 2013, 1–16. 10.5402/2013/398485

[B16] Julien C. (2006). The enigma of Mayer Waves: Facts and Models. Cardiovasc. Res. 70 (1), 12–21. 10.1016/j.cardiores.2005.11.008 16360130

[B17] Kara T. Narkiewicz K. Somers V. K. (2003). Chemoreflexes - Physiology and Clinical Implications. Acta Physiol. Scand. 177 (3), 377–384. 10.1046/j.1365-201X.2003.01083.x 12609009

[B18] Khor Y. H. Renzoni E. A. Visca D. McDonald C. F. Goh N. S. L. (2019). Oxygen Therapy in COPD and Interstitial Lung Disease: Navigating the Knowns and Unknowns. ERJ Open Res. 5 (3), 00118–02019. 10.1183/23120541.00118-2019 31544111 PMC6745413

[B19] King T. E. Pardo A. Selman M. (2019). Idiopathic Pulmonary Fibrosis. Lancet 378 (9807), 1949–1961. 10.1016/S0140-6736(11)60052-4 21719092

[B20] Kronsbein H. Gerlach D. A. Heusser K. Hoff A. Hoffmann F. Diedrich A. (2020). Testing Individual Baroreflex Responses to Hypoxia-Induced Peripheral Chemoreflex Stimulation. Clin. Auton. Res. 30 (6), 531–540. 10.1007/s10286-019-00660-6 31974825 PMC7704522

[B21] Lanfranchi P. A. Somers V. K. (2002). Arterial Baroreflex Function and Cardiovascular Variability: Interactions and Implications. Am. J. Physiology-Regulatory, Integr. Comp. Physiol. 283 (4), R815–R826. 10.1152/ajpregu.00051.2002 12228049

[B22] Lund V. E. Kentala E. Scheinin H. Klossner J. Helenius H. Sariola-Heinonen K. (1999). Heart Rate Variability in Healthy Volunteers during Normobaric and Hyperbaric Hyperoxia. Acta Physiol. Scand. 167 (1), 29–35. 10.1046/j.1365-201x.1999.00581.x 10519974

[B23] Malberg H. Wessel N. Hasart A. Osterziel K.-J. Voss A. (2002). Advanced Analysis of Spontaneous Baroreflex Sensitivity, Blood Pressure and Heart Rate Variability in Patients with Dilated Cardiomyopathy. Clin. Sci. 102 (4), 465–473. 10.1042/cs1020465 11914109

[B24] Mancia G. Grassi G. (2014). The Autonomic Nervous System and Hypertension. Circ. Res. 114 (11), 1804–1814. 10.1161/CIRCRESAHA.114.302524 24855203

[B25] Mancia G. Parati G. Pomidossi G. Casadei R. Di Rienzo M. Zanchetti A. (1986). Arterial Baroreflexes and Blood Pressure and Heart Rate Variabilities in Humans. Hypertension 8 (2), 147–153. 10.1161/01.hyp.8.2.147 3080371

[B26] Menuet C. Connelly A. A. Bassi J. K. Melo M. R. Le S. Kamar J. (2020). PreBötzinger Complex Neurons Drive Respiratory Modulation of Blood Pressure and Heart Rate. Elife 9, e57288. 10.7554/eLife.57288 32538785 PMC7326498

[B27] Minarini G. (2020). “Root Mean Square of the Successive Differences as Marker of the Parasympathetic System and Difference in the Outcome after ANS Stimulation,” in Autonomous Nervous System Monitoring-Heart Rate Variability. Editor Aslanidis T. (London: IntechOpen Press), 15–28. 10.5772/intechopen.89827

[B28] Mohammed J. Da Silva H. Van Oosterwijck J. Calders P. (2017). Effect of Respiratory Rehabilitation Techniques on the Autonomic Function in Patients with Chronic Obstructive Pulmonary Disease: A Systematic Review. Chron. Respir. Dis. 14 (3), 217–230. 10.1177/1479972316680844 28774205 PMC5720228

[B29] Mohammed J. Meeus M. Derom E. Da Silva H. Calders P. (2015). Evidence for Autonomic Function and its Influencing Factors in Subjects with COPD: A Systematic Review. Respir. Care 60 (12), 1841–1851. 10.4187/respcare.04174 26487747

[B30] Papadopoulos C. E. Pitsiou G. Karamitsos T. D. Karvounis H. I. Kontakiotis T. Giannakoulas G. (2008). Left Ventricular Diastolic Dysfunction in Idiopathic Pulmonary Fibrosis: a Tissue Doppler Echocardiographic Study. Eur. Respir. J. 31 (4), 701–706. 10.1183/09031936.00102107 18057055

[B31] Peng C. K. Havlin S. Stanley H. E. Goldberger A. L. (1995). Quantification of Scaling Exponents and Crossover Phenomena in Nonstationary Heartbeat Time Series. Chaos 5 (1), 82–87. 10.1063/1.166141 11538314

[B32] Porta A. Faes L. Bari V. Marchi A. Bassani T. Nollo G. (2014). Effect of Age on Complexity and Causality of the Cardiovascular Control: Comparison between Model-Based and Model-free Approaches. Plos One 9 (2), e89463. 10.1371/journal.pone.0089463 24586796 PMC3933610

[B33] Reulecke S. Charleston-Villalobos S. Voss A. González-Camarena R. González-Hermosillo J. Gaitán-González M. J. (2016). Orthostatic Stress Causes Immediately Increased Blood Pressure Variability in Women with Vasovagal Syncope. Comp. Methods Programs Biomed. 127, 185–196. 10.1016/j.cmpb.2015.12.005 26775735

[B34] Rico M. F. G. Urias A. P. Barquera C. S. Ochoa J. L. G. Padilla N. M. A. Meneses G. L. C. (2001). Spirometric and Gasometric Values in a Healthy Geriatric Population, at Different Altitudes above Sea Level in the Mexican Republic. Multicentric Study. Rev. Inst. Nal Enf Resp Mex 14 (2), 90–98.

[B35] Santiago-Fuentes L. M. González-Camarena R. Charleston-Villalobos S. Mejía-Ávila M. E. Reulecke S. Buendía-Roldán I. (2021). Hemodynamic Response to Low-Flow Acute Supplemental Oxygen in Idiopathic Pulmonary Fibrosis and Elderly Healthy Subjects. Heart & Lung 50 (1), 197–205. 10.1016/j.hrtlng.2020.03.025 32522419

[B36] Schulz S. Voss A. (2017). “Symbolic Dynamics, Poincaré Plot Analysis and Compression Entropy Estimate Complexity in Biological Time Series,” in Complexity and Nonlinearity in Cardiovascular Signals. Editors Barbieri R. Scilingo E. Valenza G. (Springer), 45–85. 10.1007/978-3-319-58709-7_2

[B37] Smit B. Smulders Y. M. Eringa E. C. Oudemans - van Straaten H. M. Girbes A. R. J. Wever K. E. (2018a). Effects of Hyperoxia on Vascular Tone in Animal Models: Systematic Review and Meta-Analysis. Crit. Care 22 (1), 189. 10.1186/s13054-018-2123-9 30075723 PMC6091089

[B38] Smit B. Smulders Y. M. Van der Wouden J. C. Oudemans-Van Straaten H. M. Spoelstra-de Man A. M. E. (2018b). Hemodynamic Effects of Acute Hyperoxia: Systematic Review and Meta-Analysis. Crit. Care 22 (1), 45. 10.1186/s13054-018-1968-2 29477145 PMC6389225

[B39] Stickland M. K. Fuhr D. P. Edgell H. Byers B. W. Bhutani M. Wong E. Y. L. (2016). Chemosensitivity, Cardiovascular Risk, and the Ventilatory Response to Exercise in COPD. PLoS One 11 (6), e0158341. 10.1371/journal.pone.0158341 27355356 PMC4927073

[B40] Stoller J. K. Panos R. J. Krachman S. Doherty D. E. Make B. (2010). Oxygen Therapy for Patients with COPD. Chest 138 (1), 179–187. 10.1378/chest.09-2555 20605816 PMC2897694

[B41] Van Gestel A. J. Steier J. (2010). Autonomic Dysfunction in Patients with Chronic Obstructive Pulmonary Disease (COPD). J. Thorac. Dis. 2 (4), 215–222. 10.3978/j.issn.2072-1439.2010.02.04.5 22263050 PMC3256465

[B42] Vazquez-Garcia J. C. Perez-Padilla R. (2000). Valores gasométricos estimados para las principales poblaciones y sitios a mayor altitud en México. Rev. Inst. Nal Enf Resp Mex 13 (1), 6–13.

[B43] Voss A. Schroeder R. Heitmann A. Peters A. Perz S. (2015). Short-Term Heart Rate Variability-Influence of Gender and Age in Healthy Subjects. Plos One 10 (3), e0118308. 10.1371/journal.pone.0118308 25822720 PMC4378923

[B44] Yasuma F. Hirai M. Hayano J.-i. (2001). Differential Effects of Hypoxia and Hypercapnia on Respiratory Sinus Arrhythmia in Conscious Dogs. Jpn. Circ. J. 65 (8), 738–742. 10.1253/jcj.65.738 11502051

[B45] You J. Fan X. Bi X. Xian Y. Xie D. Fan M. (2018). Association between Arterial Hyperoxia and Mortality in Critically Ill Patients: a Systematic Review and Meta-Analysis. J. Crit. Care 47, 260–268. 10.1016/j.jcrc.2018.07.014 30077082

